# The Effect of Periodontal Therapy on the Level of MMP-8 in Patients with Chronic Periodontitis

**DOI:** 10.1055/s-0041-1742132

**Published:** 2022-02-16

**Authors:** Mirlinda Sopi, Ferit Koçani, Merita Bardhoshi, Kastriot Meqa

**Affiliations:** 1 Department of Periodontology and Oral Medicine, Dentistry School, University of Pristina, Pristina, Kosovo; 2 Department of Dental Pathology and Endodontics, Dentistry School, University of Pristina, Pristina, Kosovo; 3 Department of Oro-Maxillofacial Surgery, University of Medicine, Tirana, Tirana, Albania; 4 Department of Periodontology and Oral Medicine, Dentistry School, University of Pristina, Pristina, Kosovo

**Keywords:** chronic periodontitis, diode laser, periodontal flap surgery, matrix metalloproteinase-8

## Abstract

**Objective** The complete removal of bacterial plaque and their endotoxins in deeper areas of periodontal pockets is often difficult to achieve with conventional methods such as periodontal pocket curettage. An alternative to these methods that recently gained popularity in periodontology is the diode laser, with its antibacterial effect, angiogenesis promotion as advantages.

**Materials and Methods** This study included 100 patients diagnosed with chronic periodontitis, with periodontal pockets up to 6 mm, and who were divided into two groups: patients treated with basic therapy and diode laser application, and patients treated with basic therapy and with flap surgery. The clinical periodontal evaluation was done before the treatment and 6 months after the treatment. Evaluation of matrix metalloproteinase (MMP)-8 as an inflammatory indicator in gingival exudate was done with dipstick immunoassay test before the treatment and 6 months later.

**Results** This study showed a more pronounced improvement of periodontal clinical parameters, as well as a decrease in MMP-8 values in gingival exudate in the laser-treated group compared with the surgically treated group.

**Conclusion** The results of this study are encouraging for the use of the diode laser as a noninvasive method in the treatment of periodontal pathologies.

## Introduction

Periodontitis is a chronic inflammatory disease of bacterial etiology from dental plaque. 1 Inflammation of the gingiva is a consequence of the initial lesions of the gingival epithelium caused by dental plaque microorganisms and triggering a range of reactions, such as cell infiltration, vascular changes, and changes in connective tissue elements and intercellular matter. 1 2 It is important to detect initial signs of periodontitis for preventing the progression of this disease. 3 4 Saliva is a reliable medium that reflects periodontal health and is easily accessible by identifying periodontal biomarkers. Thus, saliva is a very useful tool for monitoring not only oral but also systemic health. Periodontal tissue consists mainly of type I collagen. 5 6 7 Periodontitis is characterized by loss of connective and bone tissues, which is mainly initiated by a group of enzymes called matrix metalloproteinases (MMPs). 8 9 Therefore, laboratory detection of MMP-8 in gingival exudate enables differentiation of the inflammatory condition and degradation of periodontal tissues. 10 The MMP family is divided into six groups of proteases: collagenases (MMP-1, MMP-8, and MMP-13), gelatinases (MMP-2 and MMP-9), stromelysins (MMP-3, MMP-10, and MMP- 11), matrixes (MMP-7 and MMP-26), MMPs of the type (MMP-14, MMP-15, MMP-16, MMP-17, and MMP-12), and other unclassified MMPs, given their auxiliary contrasts. 11 MMP activities are inhibited and regulated by endogenous natural tissue inhibitors of matrix metalloproteinases (TIMPs) and α2-macroglobulin. Inequality between MMP and TIMP often results in devastating irreversible periodontal pathology. The proteolytic enzyme responsible for periodontal soft and hard tissues degeneration is the MMP-8, also known as collagenase-2 or neutrophils. 12 13 14 15 After the diagnosis, the treatment is planned, which includes all therapeutic procedures which are applied over a long period, and the success of the treatment is achieved by applying all the necessary actions. 16 17 Traditional methods of treating periodontal injuries are mainly based on the removal of pathological contents from periodontal pockets mechanically through nonsurgical interventions—scaling and root planning. 18 19 20 However, it has been argued that even after clinically successful healing, there is the possibility of reinfection by dental plaque biofilm residues. 21 22 23 24 Therefore, in the last two decades, studies on semiconductor lasers or so-called diode lasers have been deepened to achieve a better therapeutic result. 25 26 27 Clinical evidence to date points to the therapeutic advantages of using diode lasers as the first choice in the treatment of chronic periodontitis. 28 29 The diode lasers have an advantage compared with the mechanical processing of periodontal tissue, as it removes all the altered epithelium then the biological stimulatory effect normalizes cell function, increasing microcirculation, stimulating fibroblasts, osteoblasts, odontoblasts, and increasing collagen formation. 30 The laser also allows us better access to surfaces where it is anatomically difficult to reach with manual instruments. 31 The study aimed to evaluate the level of MMP-8 in chronic periodontitis before and after treatment with two therapeutic methods: 980 nm diode laser and modified Widman flap (MWF).

## Materials and Methods

This was a prospective randomized single-blinded clinical study. One hundred patients older than 18 years, with generalized (more than 30% of affected sites) stage III periodontitis (previously classified as generalized chronic periodontitis), with periodontal probing depth 4 to 6 mm deep, were included in this study. Excluded from the research were patients younger than 18 years, pregnant women, lactating women, patients currently treated with antibiotics for any other pathology, patients with addictions such as smoking, alcohol, and patients with parafunction. 26 All patients were comprehensively informed about the content and purpose of the study before being part of the research. The study protocol was approved by the Ethics Committee of the Faculty of Medicine at the University of Prishtina under the Declaration of Helsinki.

### Evaluation of Periodontal Clinical Parameters

Clinically, the condition of the periodontium was assessed using periodontal indices, such as pocket probing depth (PPD), gingival index (GI), 32 and clinical attachment level (CAL). 33 These measurements were performed using a standardized periodontal probe on six surfaces of the examined dentition: mesiovestibular, vestibular, distovestibular, mezioral, oral, and distooral. To define the extent and severity of the periodontal disease, the updated classification of periodontal and peri-implant diseases and conditions was used. 34 Evaluation of clinical parameters was done before periodontal treatment, 3 and 6 months after.

### Matrix Metalloproteinase-8 Analysis

The presence of bone destruction mediator MMP-8 was assessed by dipstick immunoassay test. The gingival exudate was taken from the periodontal pocket with the paper standing for 30 seconds, then placed in the buffer tube and the result was read after 5 minutes. Either positive or negative result was recorded. Evaluation of MMP-8 obtained from periodontal pocket exudate was done before treatment and 6 months after the treatment.

### Study Groups

Patients were randomly divided into two groups:

Group I—Patients treated with basic therapy and diode laser application.
Group II—Patients treated with basic therapy and surgical procedure with flap.

The basic therapy for both groups consisted of debridement of supragingival hard and soft deposits, as well as scaling and root planning as a nonsurgical preparation for further interventions.

Laser therapy for patients of group I was performed using a diode laser (Laser HF, Hager-Werken, Germany) with the application of laser light inside the periodontal pocket of depth up to 6 mm, and exposure of light of wavelength 980 nm with 10 mW power within 1 minute.

Standard surgical procedure using MWF for debridement of periodontal pockets was used for patients of group II. 35 It was performed by a nonauthor of this article to exclude any bias.

### Statistical Analysis

Data processing was done with the statistical package SPSS 22.0. The obtained data are presented in Tables 1 2 3 4 5 6 7 to 8 .

**Table 1:** CAL before the treatment in both groups of patients

Baseline CAL	Laser treatment	Surgical treatment
*N*	50	50
Average (mm)	4.42	4.38
SD	1.03	0.99
SEM	0.12	0.11
Minimum	2.77	2.87
Maximum	7.32	7.21

Abbreviations: CAL, clinical attachment level; SD, standard deviation; SEM, standard error of the mean.

Note: Kruskal–Wallis' test, *p* -value KW = 4.15, *p* = 0.056.

**Table 2:** CAL 6 months after the treatment in both groups of patients

CAL after 6 mo	Laser treatment	Surgical treatment
*N*	50	50
Average (mm)	3.18	3.29
SD	0.89	0.83
SEM	0.10	0.10
Minimum	2.01	2.00
Maximum	6.01	5.79

Abbreviations: CAL, clinical attachment level; SD, standard deviation; SEM, standard error of the mean.

Note: Kruskal–Wallis' test, *p* -value KW = 6.55, *p* = 0.036.

**Table 3:** PPD before the treatment in both groups of patients

Baseline PPD	Laser treatment	Surgical treatment
*N*	50	50
Average (mm)	4.18	4.14
SD	0.66	0.58
SEM	0.06	0.06
Minimum	2.24	2.46
Maximum	6.25	5.82

Abbreviations: PPD, periodontal pocket depth; SD, standard deviation; SEM, standard error of the mean.

Note: Kruskal–Wallis' test, *p* -value KW = 5.63, *p* = 0.058.

**Table 4:** PPD 6 months after the treatment in both groups of patients

PPD after 6 mo	Laser treatment	Surgical treatment
*N*	50	50
Average (mm)	2.78	2.95
SD	0.53	0.50
SEM	0.06	0.06
Minimum	2.00	2.00
Maximum	5.73	5.64

Abbreviations: PPD, periodontal pocket depth; SD, standard deviation; SEM, standard error of the mean.

Note: Kruskal–Wallis' test, *p* -value KW = 26.8, *p* < 0.0001.

**Table 5:** GI before the treatment in both groups of patients

Baseline GI	Laser treatment	Surgical treatment
*N*	50	50
Average (0–3)	2.07	2.10
SD	0.26	0.33
SEM	0.03	0.04
Minimum	1.24	1.02
Maximum	3.00	3.00

Abbreviations: GI, gingival index; SD, standard deviation; SEM, standard error of the mean.

Note: Kruskal–Wallis' test, *p* -value KW = 2.55, *p* = 0.235.

**Table 6:** GI 6 months after the treatment in both groups of patients

GI after 6 mo	Laser treatment	Surgical treatment
*N*	50	50
Average (0–3)	0.16	0.28
SD	0.40	0.45
SEM	0.04	0.05
Min	0.00	0.00
Max	2.33	2.20

Abbreviations: GI, gingival index; SD, standard deviation; SEM, standard error of the mean.

Note: Kruskal–Wallis' test, *p* -value KW = 11.0, *p* = 0.004.

**Table 7:** MMP-8 before the treatment in both groups of patients

Baseline MMP-8	Laser treatment	Surgical treatment
*N*	50 (%)	50 (%)
Negative	10 (20)	9 (18)
Positive	40 (80)	41 (82)
Total	50 (100)	50 (100)

Abbreviations: MMP-8, matrix metalloproteinase-8; SD, standard deviation; SEM, standard error of the mean.

Note: Chi-square test, *p* -value chi-square = 0.07, *p* = 0.7988.

**Table 8:** MMP-8 6 months after the treatment in groups of patients

MMP-8 after 6 mo	Laser treatment	Surgical treatment
*N*	50 (%)	50 (%)
Negative	48 (96)	40 (80)
Positive	2 (4)	10 (20)
Total	50 (100)	50 (100)

Abbreviations: MMP-8, matrix metalloproteinase-8; SD, standard deviation; SEM, standard error of the mean.

Note: Chi-square test, *p* -value chi-square = 6.06, *p* = 0.0138.

## Results

The research results show that both treatment methods have shown improvement compared with the baseline values before treatment. But the treatment with diode laser showed to be more effective in improvement of all clinical parameters, such as PPD, CAL, GI, and the level of MMP-8.

## Discussion

The key to the successful treatment of periodontal disease is early diagnosis, which aids us to prevent disease progression, thus reducing the likelihood of periodontal tissues' loss. The earlier diagnosis, the better prognosis of the disease. Following the evidence of scientific literature, worldwide experiences, and the need for less invasive treatment, this research evaluated the application of diode laser compared with the surgical method of treatment of periodontal disease and analyzed the level of MMP-8 in gingival exudate from the periodontal pocket.

In our surveyed patients before the treatment, around 80% of them appear to have the presence of MMP-8 in gingival exudate taken from the periodontal pocket, a strong correlation between clinical periodontal parameters and the presence of MMP-8 in gingival exudate. Research by Räisänen et al (2021) demonstrated the association between periodontal diagnostic clinical parameters (PPD) and the presence of MMP-8 in gingival exudate in patients with chronic periodontitis. 31

In our research, all analyzed periodontal clinical parameters improved in 6 months after laser treatment compared with patients who were treated with periodontal surgery, but with statistically important significance were PPD and CAL. In the MMP-8 assessment at 6 months, 96% of laser treatment cases were negative compared with cases treated with surgery (80%).

Khan et al (2021) evaluated the adjunct effect of 980 nm diode laser treatment of periodontal surgical therapy (MWF) for the treatment of chronic periodontitis. Periodontal surgical therapy together with laser therapy as an adjunct method led to a significant improvement of clinical parameters such as periodontal pocket depth, CAL, and better bactericidal effect 3 months after the treatment. 36

Sezen et al (2020) evaluated the clinical and biochemical efficacy of laser treatment (Er, Cr: YSGG) compared with nonsurgical periodontal treatment, in patients with periodontitis. Both treatment modalities resulted in significant improvements in clinical parameters, but laser treatment was shown to be more effective in reducing periodontal inflammation. There were no statistically significant differences in interleukin (IL)-1β and MMP-8 levels between the groups ( *p* < 0.05). 37

Deshmukh et al (2018) in their study evaluated the clinical and microbiological parameters, comparing the efficiency of periodontal treatment with diode laser and periodontal flap surgery treatment. The laser-treated group was found to have a higher decrease in the depth of periodontal pockets compared with the surgically treated group. The bactericidal effect of the laser was also significantly clearer in the reduction of periopathogens compared with the group treated with flap surgery. 38

Karthikeyan et al (2019) in their research analyzed the effect of diode laser treatment as adjunctive therapy with Kirkland flap surgery. Diode laser treatment as adjunctive therapy in Kirkland surgery has resulted in a greater reduction of clinical and microbiological parameters compared with Kirkland surgery alone, thus offering additional benefits in treating patients with chronic periodontitis. 39

Saglam et al (2014) in their research on the clinical and biochemical effects of diode laser in the treatment of periodontitis showed significant improvements in clinical parameters and reduction of the level of MMP-8 in laser-treated patients compared with the group who were treated with scaling and root planning (SRP) only. 40

Lobo and Pol (2015) investigated the adjunct effect of diode laser compared with periodontal surgical treatment, based on clinical parameters (periodontal pocket depth, CAL, gingival atrophy, plaque index, GI, and tooth mobility) in early, 3, and 6 months after treatment. Their results showed a significant improvement of all periodontal clinical parameters after the therapy, but no statistically significant difference was encountered between the two treatment groups, except for a more noticeable reduction in gingival inflammation in the laser treatment group. Generally, patients regarded the laser treatment as more acceptable. 41

## Conclusion

Concerning the periodontal clinical parameters of this research, it can be concluded that in patients treated with diode laser, there was an increase in CAL 3 and 6 months after treatment, a decrease in the depth of the periodontal pocket after 6 months, and a decrease of the GI, compared with the group treated with periodontal surgery. About the biological mediator of bone destruction, we have observed a decrease in the level of MMP-8 with a statistically significant difference in the cases treated with laser compared with the group treated with surgery. Based on these results, it would be interesting to extend the application of laser in the treatment of chronic periodontitis and apply clinical methods and protocols of this advanced technology.
